# The Effect of *Cotinus coggygria* Mouthwash on Halitosis and Oral Hygiene in Orthodontic Patients: A Randomized Clinical Trial

**DOI:** 10.3390/dj14050266

**Published:** 2026-05-04

**Authors:** Angeliki Granika, Konstantinos Karamesinis, Ioulia-Maria Mylonopoulou, Antigoni Alexiou, Iosif Sifakakis

**Affiliations:** 1 School of Dentistry, National and Kapodistrian University of Athens, 2 Thivon Str., 11527 Athens, Greece; aggelikigranika@gmail.com; 2 Department of Orthodontics, School of Dentistry, National and Kapodistrian University of Athens, 2 Thivon Str., 11527 Athens, Greece; k.karamesinis@gmail.com (K.K.); marilimyl@dent.uoa.gr (I.-M.M.); alexiou.antigoni@gmail.com (A.A.)

**Keywords:** halitosis, orthodontic treatment, mouthwash, *Cotinus coggygria*, volatile sulphur compounds, OralChroma

## Abstract

**Background/Objectives**: This study evaluated the effectiveness of *Cotinus coggygria* (Smoke Tree) Flower Water mouthwash in reducing halitosis and improving oral hygiene parameters among adolescents undergoing fixed orthodontic treatment. **Methods**: A double-blind, randomized, placebo-controlled, parallel-group clinical trial was conducted with 30 individuals undergoing treatment with fixed orthodontic appliances. Participants were allocated (1:1) into two groups: Group A received the *Cotinus coggygria* mouthwash, while Group B received the placebo mouthwash. Hydrogen sulfide (H_2_S) concentration in breath, measured by the OralChroma^TM^ II device, was the primary outcome. Secondary outcomes included dimethyl sulfide [(CH_3_)_2_S] and methyl mercaptan (CH_3_SH) levels, assessed with the same device, and oral hygiene status evaluated using the Modified Silness & Löe Plaque (PI-M) as well as the Silness & Löe Gingival (GI) indices. Normality of the data distribution was assessed using the Shapiro–Wilk test. Statistical analyses were conducted using the Mann–Whitney U test and Student’s *t*-test. **Results**: A statistically significant reduction in H_2_S levels was observed in the *C. coggygria* group compared to placebo (*p* = 0.014). Median H_2_S levels decreased from 147.00 ppb at baseline (T0) to 35.00 ppb at follow-up (T1) after 2 weeks. A statistically significant reduction in total VSC levels was also observed in the *C. coggygria* group compared to placebo (*p* < 0.001). Median total VSC levels decreased from 254.00 ppb at baseline (T0) to 105.00 ppb at follow-up (T1) after 2 weeks. No significant differences were found between groups for the other volatile sulfur compounds. A Significant improvements were noted in periodontal parameters in favor of the *C. coggygria* group. The Gingival Index decreased from 2.0 to 1.3 (*p* < 0.001; 95% CI: −0.7 to −0.2), and the Plaque Index (PI-M) decreased from 1.6 to 1.0 (*p* = 0.001; 95% CI: −0.7 to −0.3). **Conclusions**: *Cotinus coggygria* mouthwash appeared to be an effective adjunct for managing halitosis and improving oral hygiene parameters in adolescents undergoing fixed orthodontic treatment. No adverse effects were reported.

## 1. Introduction

Halitosis is defined as unpleasant or offensive breath and may result from dietary habits, inadequate oral hygiene, systemic or oral diseases, or unhealthy lifestyle factors [1]. The term derives from the Latin *halitus* (breath) and the Greek suffix -*osis*, denoting a pathological condition [2]. Halitosis is among the most common complaints in dental practice and represents the third leading reason for seeking dental care, after periodontal disease and dental caries [3]. It can significantly impair social interactions and quality of life [4], with epidemiological studies reporting a prevalence of 30–50% in the general population [5].

Oral halitosis is classified as physiologic or pathologic [6]. Physiologic halitosis occurs without detectable oral pathology, whereas pathologic halitosis is associated with oral diseases or conditions contributing to malodor. Additional contributing factors include peri-implantitis, periodontal disease, untreated carious lesions, necrotic pulp tissue, defective dental restorations, hyposalivation, mucosal lesions, and orthodontic appliances [1,7,8]. Lifestyle factors such as tobacco use, excessive alcohol consumption, and intake of odorous foods and beverages may exacerbate halitosis. At the same time, hormonal fluctuations during the menstrual cycle have also been associated with transient increases in oral malodor [7].

Oral malodor primarily results from volatile sulfur-containing compounds (VSCs), including hydrogen sulfide (H_2_S), methyl mercaptan (CH_3_SH), and dimethyl sulfide [(CH_3_)_2_S], produced through amino acid metabolism by Gram-negative oral bacteria [8]. The severity of halitosis correlates with VSC concentrations and H_2_S has been linked with increased apoptosis of gingival cells and greater vulnerability to periodontitis [9].

Objective assessment of halitosis is achieved by measuring volatile sulfur compound (VSC) concentrations in exhaled air. The dorsal surface of the tongue serves as a major reservoir for malodor-producing microorganisms, as its fissures, crypts, and papillae create favorable anaerobic conditions for VSC production. Bacterial species implicated in halitosis include *Aggregatibacter actinomycetemcomitans*, *Eikenella corrodens*, *Porphyromonas gingivalis*, *Tannerella forsythia*, *Treponema denticola*, *Fusobacterium nucleatum*, and *Prevotella intermedia* [8]. Oral malodor is likely the result of complex microbial interactions, reflected by the high species diversity observed in halitosis samples [1].

Fixed orthodontic appliances are associated with an increased risk of halitosis due to enhanced plaque retention around attachments. Surface roughness further promotes plaque accumulation and maturation, thereby facilitating malodor development. In addition, fixed appliances may limit the tongue’s self-cleansing ability by restricting the removal of food debris from dental surfaces [10].

Halitosis can be objectively assessed using halimetry, organoleptic assessment, or gas chromatography [11]. Organoleptic assessment is a simple method in which an examiner evaluates exhaled breath using a standardized scale from 0 to 5 [2]. Gas chromatography (GC) is considered the gold standard because it allows the differentiation of individual VSCs, including hydrogen sulfide, methyl mercaptan, and dimethyl sulfide. OralChroma^TM^ is a widely used portable device that combines a semiconductor gas sensor with a compact GC system [11]. Breath samples are introduced via a plastic syringe, and compounds are identified using a computerized database. Total VSC levels exceeding 150 ppb(parts per billion) indicate halitosis [11].

Management of halitosis includes mechanical approaches, such as tongue scrapers and interdental brushes, as well as chemical agents, including mouthwashes, chewing gums, and toothpastes [4]. Reducing bacterial biofilm formation and food debris retention decreases the number of VSC-producing microorganisms. Historically, herbal products were widely used for disease treatment, and several chemical agents—including mastic-based products, green tea, mushrooms, probiotics, and plant extracts—have demonstrated VSC-reducing properties and efficacy in managing oral malodor [4,12,13]. A recent trial concluded that a mastic toothpaste may significantly reduce the VSC levels in orthodontic patients further supporting the role of plant extracts in oral malodor management [13].

*Cotinus coggygria* Scop. (Anacardiaceae), commonly known as the smoke tree, has long been recognized for its medicinal properties [14]. In traditional medicine, it has been used for its antiseptic, anti-inflammatory, and antihemorrhagic effects. Additionally, *C. coggygria* (CC) exhibits wound-healing, antioxidant, and antibacterial activities, and its antimicrobial properties have led to its use as an oral rinse for treating abscesses and inflammatory conditions of the oral cavity [14,15]. The effect of CC Flower Water mouthwash on halitosis and on oral hygiene indexes in patients undergoing orthodontic treatment has not been previously investigated.

Objectives:

The aim of this prospective trial was to evaluate the effect of CC Flower Water mouthwash (Natural smoke tree Hydrolina, Ina Essentials^®^ Ina Trade LTD, Panagyurishter, Bulgaria) in reducing halitosis, plaque accumulation, and gingival inflammation in adolescent patients undergoing orthodontic treatment with conventional fixed labial appliances. The null hypothesis was that the use of CC Flower Water mouthwash and placebo mouthwash during orthodontic treatment would not affect objective VSC levels and specific patients’ oral hygiene parameters.

## 2. Materials and Methods

### 2.1. Patient and Public Involvement, Trial Design, Trial Setting and Eligibility Criteria

This was a randomized, placebo-controlled, double-blinded trial with two parallel groups and a 1:1 allocation ratio. At the beginning of the clinical trial, the study details were explained to the patients. Parents or legal guardians provided informed consent before the participants entered the trial. This clinical trial was registered at ClinicalTrials.gov (NCT07254819, registration date: 19 November 2025) and conducted in accordance with the SPIRIT Statement and CONSORT guidelines. This clinical trial was carried out in compliance with the ethical principles derived from the Declaration of Helsinki, and in accordance with Good Clinical Practice guidelines and all applicable regulatory requirements. Moreover, this study was approved by the Ethics Committee of the School of Dentistry, National and Kapodistrian University of Athens (680/3 February 2025). The trial included 30 patients undergoing orthodontic treatment with conventional labial fixed appliances, treated by residents in the postgraduate clinic of the Department of Orthodontics of the National and Kapodistrian University of Athens, Greece (NKUA). The survey period was 2 weeks, and eligibility for participation was determined based on the following inclusion criteria: (1) Patients between 13 and 18 years of age, (2) with fixed conventional labial appliances on both dental arches, (3) stainless steel brackets or bands (same manufacturer) on at least 24 teeth for over 4 months prior to recruitment, (4) bands on the first molars, (5) expected duration of the remaining orthodontic treatment more than 1 month, (6) last extraction at least 2 months before the start of the experiment (in extraction cases), (7) good general health, and (8) baseline total VSC levels greater than 150 ppb. Patients with active caries, periodontitis (periodontal pockets >3 mm), allergy to CC (smoke tree), dental fluorosis or dysplasia, syndromic conditions or abnormalities affecting the craniofacial complex, mental health issues, tobacco use, chlorhexidine or other mouthwash use in the previous three weeks, taking antibiotics during the last 2 months, and enrolling in other ongoing clinical trials were excluded.

### 2.2. Changes to Trial Protocol

No modifications to the study protocol were made after the trial began.

### 2.3. Intervention and Comparator

Upon recruitment, the patients were equally and randomly allocated to:

- group A (*n* = 15) the CC Flower water mouthwash containing 100% Smoketree Hydrolate (NATURAL SMOKE TREE HYDROLINA, INA ESSENTIALS^®^) or
- group B *(n* = 15) the placebo mouthwash (from the same manufacturer) containing 99.8% water and 0.2% food coloring.

NATURAL SMOKE TREE HYDROLINA is produced through primary steam distillation of freshly cut plants with the chemical name CC Leaf Extract. The hydrolate contains 100% natural plant water with no added fragrances, colorants, or other ingredients. The mouthwash contains 0.019% (m/m) essential oils and 0.94% (*v*/*v*) Ethyl alcohol. The product’s identity is established by its INCI declaration, while its quality is confirmed through appropriate quality documentation.

Due to differences in composition, active and placebo mouthwashes may have differed in organoleptic properties, including taste, aroma, and mouthfeel. For this reason, both formulations were provided in identical packaging with similar appearance and labeling, and participants were not informed about the specific characteristics of each product to ensure visual blinding of participants and investigators.

The participants were instructed to rinse their mouths following brushing, with 10 mL of mouthwash for 30 s, in the morning and before going to bed, for 14 days. They were also advised to follow their usual oral hygiene habits during the study period to avoid altering outcomes through improved routines. All assessments were carried out in the morning, with a minimum interval of three hours after brushing. Participants were instructed to avoid eating or drinking anything other than water for 3 h prior to the test and to avoid consuming odiferous foods, including garlic, onion, and spices, 24 h before the assessment [3]. Modified plaque index (PI-M), Gingival Index (GI) and VSC levels were assessed at baseline (T0) and after 2 weeks of mouthwash use (T1). Adverse events were systematically monitored throughout the study. At follow-up visit, participants were actively questioned regarding the occurrence of any side effects, including oral mucosal irritation, taste disturbances, allergic reactions, xerostomia or any other unexpected symptoms. Any adverse effects were planned to be recorded and evaluated.

### 2.4. Outcomes

The primary outcome of the study was the concentration of hydrogen sulfide (H_2_S) in exhaled breath, measured using a portable gas chromatograph (OralChroma^TM^ II, (NOVATRONIC Deutschland GmbH, Bergisch Gladbach, Germany). Secondary outcomes included methyl mercaptan (CH_3_SH) and dimethyl sulfide [(CH_3_)_2_S] concentrations, assessed using the same device. Breath sampling and analysis were performed according to a standardized protocol. All measurements were conducted in the morning under controlled conditions. Participants were instructed to refrain from eating, drinking (except water), performing oral hygiene procedures, or using any oral products for at least 3 h before the assessment, and to avoid consumption of odorous foods 24 h before measurement. For breath collection, participants were asked to keep their mouths closed and breathe nasally for 1 min to allow for accumulation of intraoral gases. Subsequently, a sterile disposable 1 mL syringe was inserted into the oral cavity, and approximately 0.5 mL of mouth air was immediately injected into the OralChroma^TM^ device. The device separates VSCs through gas chromatography and quantifies each compound based on retention time and peak area using an internal calibration system. Concentrations of H_2_S, CH_3_SH, and (CH_3_)_2_S were automatically calculated and expressed in parts per billion (ppb). To ensure reliability, measurements were performed under identical environmental conditions and by calibrated examiners. Where necessary, duplicate measurements were obtained and averaged.

Neither the Modified Silness and Löe Plaque Index nor the Silness and Löe Gingival Index reflects plaque distribution patterns in patients with orthodontic appliances. To address this limitation, each tooth was segmented into four areas (mesial, distal, gingival, and incisal) in relation to the orthodontic bracket [16]. Plaque accumulation in each area was assessed using the original index scores (0–3) (Table 1). The scores from the four regions were then summed up to produce a total score per tooth, ranging from 0 to 12. This adapted index is considered particularly suitable for orthodontic patients, as it reflects the typical plaque distribution around brackets and offers improved discriminatory capacity between categories [16,17].

Assessment of plaque in the gingival area was not always feasible because mild inflammation of the soft tissues and gingival overgrowth—frequently observed during orthodontic treatment—could obscure this region. In addition, brackets may have been positioned very close to the gingival margin on partially erupted teeth, such as second premolars and second molars. In these cases, when the bracket was near the soft tissues, plaque in the gingival region was not recorded. Consequently, for these teeth, the maximum possible score was 9 rather than 12 (number of assessed surfaces × 3).

To standardize scoring, the total plaque score for each tooth was divided by its respective maximum possible score to calculate a mean tooth score. The individual’s overall score was accordingly determined by averaging the scores of all assessed teeth. Only the labial surfaces of bonded teeth were assessed. For the modified plaque index (PI-M), four regions on the labial surface of each bonded tooth would be evaluated as previously described, while banded molars were excluded from measurement (Table 1). For each participant, the mean GI score was calculated using the three designated areas of the buccal surface (distal, cervical, and mesial) of each tooth (Table 2) [18]. Banded molars were included in the examination [18].

### 2.5. Sample Size

The sample size was calculated using G*Power software (version 3.1.9.7, Heinrich Heine University Düsseldorf, Germany), based on an expected mean difference in VSC levels of 50 ppb and a standard deviation of 60 [4]. A two-sided test was applied, with a significance level (α) of 0.05, a 95% confidence interval, and 80% statistical power. The minimum required sample size was estimated at 12 participants per group. To strengthen the robustness of the study and account for a potential dropout rate (approximately 20%), the sample size was increased to 15 participants per group.

### 2.6. Randomization (Sequence Generation, Allocation Concealment Mechanism, Implementation, Blinding)

Stratified randomization by sex was used to allocate participants equally (1:1) to either Group A (CC mouthwash) or Group B (placebo mouthwash). Two separate random sequences of 15 letters (A or B) were generated for males and females using the List Randomizer service (www.random.org) by an investigator not involved in participant recruitment or outcome assessment. The generated random sequences were saved and archived prior to participant enrollment to ensure reproducibility and allow verification of the randomization process. The letters were printed and securely sealed in opaque envelopes, each clearly numbered in sequence: M1–M15 for men and F1–F15 for women, to ensure allocation concealment. Participant enrollment was performed by the investigators, while group assignment was carried out by an independent individual who opened the envelopes in sequential order and provided the corresponding intervention. Both participants and outcome assessors were blinded to group allocation. To maintain blinding, the active and placebo mouthwashes were identical in packaging, appearance, and labeling.

### 2.7. Statistical Methods

Inter- and intra-observer reliability for VSC measurements was assessed using the ICC. To evaluate intra-observer error, the principal investigator repeated the VSC measurements in 10 enrollees. To assess inter-observer reliability, both the principal investigator and the second author repeated the measurements on 10 patients. Normality of the data distribution was assessed using the Shapiro–Wilk test. VSC level data were not normally distributed; therefore, non-parametric tests (Mann–Whitney U test) were used for between-group comparisons. In contrast, oral hygiene indices met the assumptions of parametric analysis, including normal distribution and homogeneity of variance, and were analyzed using the independent samples Student’s *t*-test. Statistical significance was set at *p* < 0.05, with a 95% confidence interval. Blinded investigators evaluated all results. IBM SPSS Statistics 23.0 was used for all statistical tests.

## 3. Results

### 3.1. Participant’s Flow, Recruitment

**Figure 1:**
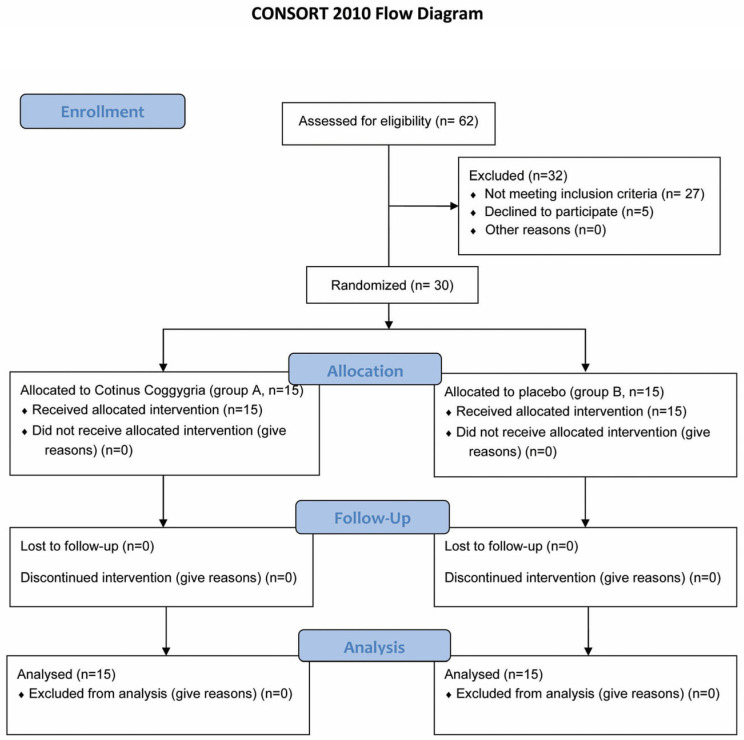
The CONSORT 2010 Flow Diagram.

Sixty-two patients were assessed for eligibility, and 30 patients who met the inclusion criteria participated in the study. Patient recruitment began in December 2025, and all participants were recruited and underwent baseline assessment (T0) on the same day, ensuring a uniform 14-day follow-up interval until the final evaluation (T1), which was completed after 2 weeks (Figure 1).

### 3.2. Intervention and Comparator Delivery

The intervention was delivered as planned by a person not involved in the trial measurements and results. All participants self-reported performing mouthwash procedures twice daily. Adherence to the protocol was also verified by parental confirmation and the volume of returned mouthwash. Concomitant care was similar between groups. No additional interventions relevant to the study outcomes were performed.

### 3.3. Baseline Data

The baseline measurements are depicted in Table 3. The mean age (±standard deviation) was 14.70 years (±1.79) for the CC group (group A) and 14.79 years (±1.60) for the placebo group (group B).

### 3.4. Numbers Analyzed, Outcomes and Estimation

Intra-observer and inter-observer ICCs were 0.74 and 0.77, respectively. VSC data did not follow a normal distribution. In the CC group (Group A), median total VSC levels decreased from 254.00 ppb to 105.00 ppb. Specifically, H_2_S levels declined from 147.00 ppb to 35.00 ppb; CH_3_SH from 52.00 ppb to 13.00 ppb; and (CH_3_)_2_S from 39.00 ppb to 28.00 ppb. In the placebo group (Group B), median total VSC levels dropped from 229.00 ppb to 198.00 ppb; H_2_S concentrations decreased from 180.00 ppb to 137.00 ppb; whereas CH_3_SH levels rose from 24.00 ppb to 25.00 ppb; and (CH_3_)_2_S levels increased from 14.00 ppb to 28.00 ppb. The treatment demonstrated statistical significance for the primary outcome (H_2_S), as well as for the total VSCs levels. Group A’s PI-M mean score decreased from 1.62 to 1.03, and Group B’s PI-M mean score dropped from 1.63 to 1.53. Group A’s mean GI score decreased from 1.95 to 1.32, and Group B’s GI mean score dropped from 2.02 to 1.81. For GI and PI scores, the treatment effect was statistically significant, in favor of the CC group (*p* = 0.001 and *p* < 0.001, respectively) (Table 3).

### 3.5. Harms

No adverse events were reported in either group during the study period. Participants did not report any oral mucosal irritation, taste disturbances, allergic reactions, or other unexpected side effects following the use of either the CC or placebo mouthwash.

### 3.6. Ancillary Analyses

No ancillary analyses were performed.

## 4. Discussion

This study is the first randomized clinical trial to evaluate the efficacy of CC mouthwash in managing halitosis among adolescents with fixed orthodontic appliances. However, given the limited sample size (*n* = 30) and short follow-up period (14 days), the findings should be interpreted with caution and considered preliminary and hypothesis-generating rather than definitive. Within these limitations, the results demonstrated a statistically significant reduction in hydrogen sulfide (H_2_S) and in total volatile sulfur compounds (VSC) levels in the intervention group compared to placebo, while no significant differences were observed for methyl mercaptan (CH_3_SH) and dimethyl sulfide ((CH_3_)_2_S). An important finding of the present study is that H_2_S levels also decreased in the placebo group, from 180.00 ppb at baseline to 137.00 ppb at follow-up, representing a reduction of approximately 24%; however, this decrease did not reach statistical significance. This indicates that part of the observed improvement may be attributed to a placebo effect, natural variability, or regression to the mean. Additionally, participation in a clinical trial may have influenced patients’ oral hygiene behaviors despite instructions to maintain their usual routines. Therefore, although the reduction in H_2_S was significantly greater in the CC group, these factors should be considered when interpreting the magnitude of the treatment effect. The lack of statistically significant between-group differences for CH_3_SH and (CH_3_)_2_S warrants further consideration. One possible explanation relates to the distinct biological origins and distribution of these compounds. H_2_S is primarily associated with tongue coating and overall oral biofilm activity. CH_3_SH is more closely linked to periodontal pockets and tissue inflammation, whereas (CH_3_)_2_S has been associated not only with intraoral but also extraoral or systemic sources [6,8,9]. Given that the study population consisted of adolescents without periodontitis, the baseline contribution of CH_3_SH may have been limited, reducing the potential for detectable changes. Thus, the short duration of the intervention (14 days) may have been insufficient to affect compounds originating from more complex or less accessible reservoirs. In addition, significant improvements in plaque accumulation and gingival inflammation were observed in the CC group. The use of the OralChroma^TM^ device, which exhibits high sensitivity particularly for H_2_S detection, strengthens the reliability of the primary outcome assessment [11].

Halitosis is primarily caused by bacterial activity, leading to the production of VSC, including H_2_S, (CH_3_)_2_S and CH_3_SH. A recent study evaluated a mouthwash containing low concentrations of chlorhexidine and zinc and demonstrated a significant reduction in H_2_S levels, highlighting its central role in oral malodor and the effectiveness of combining antimicrobial and VSC-neutralizing agents [19]. These findings are in agreement with the present study, where a significant reduction in H_2_S was also observed. However, other VSCs, such as (CH_3_)_2_S and CH_3_SH, may also contribute to halitosis [11]. In a recent study, the use of a mastic mouthwash reduced H_2_S levels; however, the levels of the other VSCs did not differ between the two groups [12]. Another randomized clinical trial concluded that a ClO_2_ mouthwash significantly reduced VSCs levels [4]. In the present trial, (CH_3_)_2_S and CH_3_SH levels decreased in the CC group, whereas a slight increase was observed in the placebo group. Nevertheless, no statistically significant differences were detected between the two groups for these compounds.

Managing halitosis also requires addressing both periodontal and cariogenic pathogens [4,8]. Conventional mouthwashes often rely on antibacterial agents, such as chlorhexidine, which, despite their efficacy, act non-selectively on the oral microbiota and may disrupt microbial balance with prolonged use [20]. In addition, their long-term use is associated with adverse effects, including dysgeusia, extrinsic tooth staining, and mucosal irritation [4,8], while alcohol-containing formulations may contribute to xerostomia [21]. These limitations underscore the necessity for safer, less cytotoxic therapeutic alternatives [22]. In this context, natural extracts have attracted increasing interest due to their antimicrobial properties. Notably, a randomized, double-blind, placebo-controlled study by Kim et al. [21] demonstrated that a mouthwash containing *Lespedeza cuneata* extract significantly reduced halitosis-associated oral bacteria, supporting the potential of plant-derived agents in oral malodor management.

CC is a potent source of bioactive essential oils and extracts with diverse therapeutic applications [15]. Traditionally used as an infusion, its leaves possess antiseptic, anti-inflammatory, and wound-healing properties [22]. Specifically, leaf-derived essential oils effectively inhibit Gram-positive bacteria and fungi [15]. The high concentrations of polyphenols, flavonoids, and tannins found across the plant’s anatomy (shoots, flowers, leaves, and stems) further contribute to its antimicrobial and cytotoxic profile [22]. Historically, these properties have made CC preparations effective oral rinses for treating dental abscesses and oral inflammation [14,15]. Notably, ethyl acetate and acetone extracts demonstrate robust activity against both Gram-positive and Gram-negative strains—the latter of which produce malodorous hydrogen sulfide H_2_S from cysteine [8,15,22,23]. These bioactive compounds may also provide a mechanistic explanation for the findings of the present study. In particular, polyphenols, flavonoids, and tannins may inhibit the growth and metabolic activity of anaerobic Gram-negative bacteria involved in halitosis, such as *Porphyromonas gingivalis* and *Fusobacterium nucleatum*, which generate hydrogen sulfide through the degradation of sulfur-containing amino acids [8,15,22]. Furthermore, tannins may reduce bacterial adhesion and biofilm formation, thereby limiting volatile sulfur compound production [22]. This mechanism may explain the significant reduction in H_2_S levels observed in the CC group.

It should be noted, however, that the formulation used in the present study was a plant hydrolate containing a relatively low concentration of essential oils (0.019%), which differs from the more concentrated extracts and essential oils described in the literature [15,22]. Therefore, the antimicrobial mechanisms discussed above are inferred from studies using higher concentrations of bioactive compounds and may not be directly comparable. Nevertheless, hydrolates may retain biologically active components in lower concentrations, potentially contributing to the observed clinical effects. Further studies are needed to clarify the specific mechanisms of action and the active constituents responsible for the reduction in volatile sulfur compounds.

The oral hygiene indices significantly improved after the two-week use of CC mouthwash among the orthodontic patients of the present study. Another systematic review and meta-analysis comparing curcumin versus chlorhexidine mouthwashes in controlling plaque and gingivitis revealed equivalent efficacy of both mouthwashes in reducing plaque and gingivitis [24]. However, a recent clinical trial investigating the effect of a mastic mouthwash on periodontal parameters had no statistical difference between the mastic mouthwash and the placebo group [12]. The present study suggests that CC may serve as a plant-based alternative for maintaining oral hygiene parameters during orthodontic treatment.

Another way to assess halitosis is through subjective measurement via questionnaires. A recent clinical trial demonstrated no differences in the subjective evaluation between two groups of patients [12]. In another randomized trial, that evaluated the effect of a mouthwash containing Lespedeza cuneata extract on halitosis, the level of subjective improvement was significantly greater in the experimental group compared to the control (saline-gargle) group [21].

### 4.1. Limitations

The current study presents some limitations. The relatively small sample size and the short follow-up period limited the ability to assess the long-term effects and the potential adverse effects of this mouthwash. Although participants were instructed to maintain their usual oral hygiene practices, variations in compliance may have introduced confounding factors. Potential concerns regarding blinding credibility should also be acknowledged, as organoleptic differences between the active and placebo mouthwashes may have affected participant perception. Furthermore, microbiological analyses were not performed to directly assess changes in the oral microbiota. Microbial profiling at baseline could be a meaningful direction for future studies as microorganisms are significant contributors to halitosis [4,8]. Moreover, no patient-reported subjective evaluation was performed, which limits the assessment of perceived improvement in halitosis. The age range of the participants may have affected this measurement, as adolescents tend to underestimate their own oral malodor due to the strong emphasis on social acceptance [12].

Cysteine challenge is a valuable tool for evaluating the ability of the oral microorganisms to produce malodor [23]. Thus, the absence of this intervention prior to the present gas chromatography analysis represents an additional methodological limitation. Moreover, the CC group exhibited higher baseline total VSC levels compared to the placebo group (254 vs. 229 ppb), despite randomization. This may introduce regression to the mean, potentially contributing to the observed reduction. However, the placebo group did not show a similar magnitude of decrease, suggesting that the treatment effect cannot be solely explained by this phenomenon. Nevertheless, adjustment for baseline values would strengthen the analysis and should be considered in future studies.

### 4.2. Generalizability

The present study was conducted at a single institution and included only adolescent participants with fixed labial orthodontic appliances, which may limit the generalizability of the findings. Results may vary in the adult population because of differences in patient compliance or variations in oral microbial flora.

## 5. Conclusions

Within the limitations of this randomized clinical trial, CC mouthwash resulted in a statistically significant reduction in H_2_S and in total VSC levels compared to placebo in adolescents undergoing orthodontic treatment with fixed appliances. No significant between-group differences were observed for CH_3_SH and (CH_3_)_2_S. In addition, plaque and gingival indices showed a statistically significant reduction in the CC group compared to placebo. The intervention was well tolerated, with no adverse effects reported. Further studies with broader populations, including people with periodontitis or systemic conditions that provoke malodor, as well as extended follow-up and microbial assessments are warranted to confirm these findings.

## Figures and Tables

**Table 1:** Plaque index [18].

0	No plaque
1	Thin plaque attached to the gingival margin and adjacent areas of the tooth. Plaque is only visible after applying a revealing solution or using a probe on the tooth surface
2	Moderate accumulation of soft plaque deposits in the gingival sulcus or plaque on the gingival margin and adjacent areas of the tooth visible to the naked eye
3	Abundant soft plaque deposits in the gingival sulcus or plaque on the gingival margin and adjacent areas of the tooth

**Table 2:** Gingival index [18].

0	Normal gums
1	Mild inflammation, slight change in color and distinct change in texture, no bleeding upon probing
2	Moderate inflammation, moderate redness, and swelling, bleeding upon probing
3	Severe inflammation, visible swelling, ulceration, bleeding upon probing, and/or spontaneously

**Table 3:** H_2_S, CH_3_SH, (CH_3_)_2_S, total VSCs levels and periodontal indices scores at T0 and T1 for the *C. coggygria* group (group A) and the placebo group (group B) (median and mean values, IQR and SD, effect sizes).

		A: *C. coggygria*		B: Placebo			
		Median	IQR	Median	IQR	*p*-value ^∗^	*U*
H_2_S	T0	147.00	208.00	180.00	321.00		
	T1	35.00	155.00	137.00	198.00	0.014	43.50
CH_3_SH	T0	52.00	80.00	24.00	46.00		
	T1	13.00	31.00	25.00	42.00	0.106	124.00
(CH_3_)_2_S	T0	39.00	104.00	14.00	169.00		
	T1	28.00	153.00	28.00	153.00	0.056	101.50
Total VSCs	T0	254.00	240.00	229.00	227.00		
	T1	105.00	120.00	198.00	164.00	<0.001	235.00
		Mean	SD	Mean	SD	*p*-value ^∗∗^	*t*
PI-M	T0	1.62	0.30	1.63	0.31		
	T1	1.03	0.21	1.53	0.39	<0.001	−4.478
GI	T0	1.95	0.27	2.02	0.32		
	T1	1.32	0.21	1.81	0.36	0.001	−3.592

The significance of differences between groups was assessed using the Mann–Whitney *U* test (*p*-value ^∗^) and Student’s *t*-test (*p*-value ^∗∗^). IQR, interquartile range; SD, standard deviation; H_2_S, hydrogen sulfide; CH_3_SH, methyl mercaptan; (CH_3_)_2_S, dimethyl sulfide, VSCs, volatile sulfur compounds.

## Data Availability

Data available on request due to privacy/ethical restrictions.

## Abbreviations

The following abbreviations are used in this manuscript:

ppb	parts per billion
CC	*Cotinus coggygria*
GC	chromatography
GI	Silness and Löe Gingival Index
PI-M	Modified Silness and Löe Plaque Index VSCs
VSCs	Volatile sulphur compounds
H_2_S	hydrogen sulfide
CH_3_SH	methyl mercaptan
(CH_3_)_2_S	dimethyl sulphide
*P. gingivalis*	*Porphypomonas gingivalis*
ICC	Intra-class correlation coefficient
